# Editorial: Machine learning algorithms and software tools for early detection and prognosis of schizophrenia

**DOI:** 10.3389/fncom.2026.1966648

**Published:** 2026-09-09

**Authors:** Amit Singhal, Megha Agarwal, Animesh Kumar Paul, Bishal Lamichhane

**Affiliations:** 1 Department of Electronics and Communication Engineering, Netaji Subhas University of Technology, New Delhi, Delhi, India; 2 Department of Electronics and Communication Engineering, Jaypee Institute of Information and Technology, Noida, India; 3 Department of Computing Science, University of Alberta, Edmonton, AB, Canada; 4 Alberta Machine Intelligence Institute, Edmonton, AB, Canada; 5 Department of Psychiatry and Behavioral Health, Houston Methodist Academic Institute (HMAI), Houston Methodist, Houston, TX, United States; 6 Department of Electrical and Computer Engineering, Rice University, Houston, TX, United States

**Keywords:** artificial intelligence, early prediction, explainability (XAI), machine learning, schizophrenia

## Introduction

Schizophrenia and related psychotic disorders exhibit complex neurobiological mechanisms with variable disease characteristics. Early diagnosis currently relies on clinical assessments alone, since validated biomarkers and generalized prediction models are limited. Recent advances in machine learning (ML) have created a potential to analyze diverse information, including clinical assessments, neurophysiological recordings, laboratory measures, speech, digital phenotyping, and facial behavior for early detection and prognosis. However, rigorous validation and explainability are needed to improve the robustness and clinical utility of these ML-based models.

This Research Topic includes 13 contributions: 10 original research articles, two brief research reports, and one study protocol. These contributions investigate the early prediction of psychosis risk, role of candidate biomarkers, objective assessment of behavior and cognition, choice of clinically relevant outcomes, and development of clinically interpretable models for decision support. Collectively, these studies demonstrate the relevance of computational psychiatry to schizophrenia management and highlight the challenges of translating ML-derived models into clinical practice. Early identification and outcome stratification remain primary goals of computational psychiatry. Some studies in this Research Topic examined whether complementary clinical, cognitive, behavioral, and neurophysiological information can provide a more comprehensive basis for risk assessment than a single data source alone. They also emphasize that predictive performance within the original study cohort is insufficient and generalizability requires independent external validation.

Smucny et al. evaluated whether ML models trained on the demographic and clinical data from multicenter cohorts for predicting conversion to psychosis generalize to an independent cohort. Although all evaluated ML models achieve better than chance prediction accuracy in the new cohort, the model performance was substantially lower than within-cohort results and did not reach a level suitable for clinical application. Singh et al. proposed the neural-PRISM framework, which learns joint representations from electroencephalography, functional near-infrared spectroscopy, and facial-behavioral recordings obtained during live face-to-face interactions. In leave-one-subject-out validation on 33 participants, the multimodal framework improved the classification of first-episode psychosis. The classifier-derived scores were moderately correlated with Global Assessment of Functioning (GAF) and positive Positive and Negative Syndrome Scale(PANSS) scores but only weakly with negative PANSS scores. Nagasawa et al. examined clinical outcomes (four ordered categories: remission, symptomatic, prodromal progression, and psychotic) among individuals with at-risk mental states and identified baseline clinical, functional, and electrophysiological factors associated with less favorable outcomes. This work extends the ML-based modeling paradigm in schizophrenia beyond the conventional transition vs. non-transition dichotomy. Together, these studies illustrate a shift from narrowly defined diagnostic tasks based on a limited modality toward multimodal characterization, clinically meaningful outcomes, and explicit evaluation of model generalizability.

Objective assessment of cognition, thought disorder, and communication represents another area where computational methods may complement clinician observations and patient interviews. Digital tasks (behavioral tests) and ML can capture cognitive, linguistic, and behavioral characteristics that are difficult to quantify consistently using traditional approaches. In a cohort of 27 young people with early-onset psychosis, Zakowicz et al. reported the feasibility of using neuropsychological measures and antipsychotic medication load to classify formal thought disorder. Wu et al. evaluated a virtual reality game, termed as “Fruit Pioneer,” that provides a platform for cognitive assessment. Game measures were analyzed to differentiate between the schizophrenia and healthy-control groups. These measures were accompanied by high immersion and minimal simulator sickness, although broader validation is required. Liu J. et al. presented a study protocol discussing the prospective collection of speech and language data in a Chinese-speaking population and the planned use of ML and natural language processing to classify schizophrenia-spectrum disorders, examine progression over time, and estimate the severity of disorder. These contributions elucidate the potential of speech analytics, virtual reality, and ML-assisted neuropsychological assessment for scalable measurement, keeping in view the need to extend feasibility studies and protocols for larger populations to demonstrate clinical effectiveness.

The search for clinically accessible biomarkers for schizophrenia has also witnessed some recent advances. Unlike specialized laboratory techniques or imaging methods, some studies focus on routinely collected data, which could reduce the cost and implementation barriers. Ogur et al. used routine hematological and biochemical measures, Gray Wolf optimization, and cross-validated ML models to distinguish schizophrenia from healthy controls in a retrospective case-control dataset. They obtained high prediction accuracy in differentiating schizophrenia from healthy controls; however, external validation remains necessary. Chen et al. developed highly predictive ML models to identify clinically diagnosed schizophrenia among people living with HIV using routinely collected blood biomarkers. These results are encouraging but need further validation owing to the small retrospective sample sizes used for analysis and the limited consideration of confounders, such as current medications that affect biomarkers. Sierakowska et al., in a non-ML case-control study, found lower serum neuron-specific enolase (NSE) concentrations in schizophrenia than in controls. However, correlations of NSE with positive and negative symptom severity were not statistically significant. Liu S. et al. applied explainable ML to multicenter laboratory data from first-admission patients to predict future impulsivity in schizophrenia within 1 week of admission. Combining routine biomarkers with clinical information improved risk prediction of the developed ML model in an independent external cohort. Collectively, these studies support continued investigation of accessible candidate biomarkers and interpretable models, but they do not yet establish validated diagnostic or prognostic tests for routine psychiatric care.

Some studies extended ML applications beyond diagnosis to inpatient safety and physical-health management, where timely risk identification could support closer monitoring or individualized care. Meng et al. developed a hierarchical model combining baseline clinical characteristics with longitudinal nursing observations to predict violent behavior among hospitalized patients with schizophrenia. Similarly, Liang et al. integrated admission information with repeated nursing assessments to identify individuals at high risk of self-harm or suicidal behavior. In both retrospective studies, integrating static and dynamic information improved discrimination compared with using either feature set alone, but prospective and cross-site evaluation is crucial before deployment. Extending predictive modeling beyond psychiatric symptoms, Li et al. showed that cognitive performance, together with routine clinical characteristics, can effectively stratify metabolic risk in schizophrenia. Random forest showed the best overall performance, whereas the support vector machine better identified minority classes, illustrating that the model choice should reflect the clinical objective and class distribution. Taken together, these studies illustrate the potential value of routinely collected longitudinal observations and clinical features for decision support in schizophrenia management, while reinforcing the notion that such tools should complement, but not replace, the clinical judgment.

## Conclusion

This Research Topic demonstrates the expanding role of computational approaches in early risk assessment, objective symptom and cognitive measurement, candidate biomarker discovery, inpatient safety, and metabolic health evaluation in schizophrenia. The studies highlight that strong performance in small samples may not assert clinical readiness for deployment of the computational models. Meaningful progress can be achieved by a close integration of analytical methods and clinical expertise, paired with continued validation in practical settings. There exist many challenges before the clinical adoption of ML-based computational models in assisting early detection of schizophrenia and prognosis modeling. Common limitations include modest sample sizes, class imbalance, possible medication-related confounding, and robust independent validation. Future research should prioritize multicenter evaluation, model calibration, transparent reporting, clinical utility assessment, and external validation across populations to produce sufficient evidence that the computational model improves real-world clinical decision-making. Multimodal and longitudinal data may further improve the characterization of heterogeneous disease trajectories. We hope that this Research Topic will encourage collaboration among neuroscientists, psychiatrists, computer scientists, engineers, and data scientists to develop computational tools that are robust, trustworthy, and useful in real-world scenarios.

